# Prenatal Depression and Symptom Severity by Maternal Race and Ethnicity

**DOI:** 10.1001/jamanetworkopen.2025.0743

**Published:** 2025-03-13

**Authors:** Kendria Kelly-Taylor, Sara Aghaee, Joshua Nugent, Nina Oberman, Ai Kubo, Elaine Kurtovich, Charles P. Quesenberry, Ayesha C. Sujan, Kathryn Erickson-Ridout, Mibhali M. Bhalala, Lyndsay A. Avalos

**Affiliations:** 1 Division of Research, Kaiser Permanente Northern California, Pleasanton, California; 2 Stanford University School of Medicine, Stanford, California; 3 The Permanente Medical Group, Kaiser Permanente Northern California, Oakland; 4 Kaiser Permanente Redwood City Medical Center, Redwood City, California; 5 Department of Health Systems Science, Kaiser Permanente Bernard J. Tyson School of Medicine, Pasadena, California

## Abstract

This cross-sectional study compares the prevalence and risk for diagnosed and undiagnosed prenatal depression among pregnant individuals of different races and ethnicities.

## Introduction

Prenatal depression has been associated with adverse outcomes, including preterm birth and postpartum depression, and disproportionately affects racial or ethnic minoritized pregnant individuals. Black, Hispanic, and Asian individuals may experience more severe depression symptoms yet be less likely to engage in treatment, possibly due to clinical underdiagnosis.^1^ Prior research has often aggregated racial and ethnic groups, potentially masking important within-group differences and cultural nuances.^2^ To address these gaps, we examined differences in prenatal depression diagnoses (PDDs), self-reported symptoms of moderate to severe depression, and undiagnosed depression among a large cohort of racially and ethnically diverse pregnant individuals screened for depression.^3^

## Methods

This population-based cross-sectional study was conducted among Kaiser Permanente Northern California (KPNC) members aged 15 to 45 years with a singleton live birth (January 1, 2013-December 31, 2019) who had at least 1 KPNC prenatal care visit and self-reported race and ethnicity data. KPNC and California State Institutional Review Boards approved this study and waived informed consent because patient privacy and records confidentiality requirements were met. We followed the STROBE reporting guideline.

Race and ethnicity were ascertained from California State birth records and KPNC databases. Twenty racial and ethnic groups were identified for analysis (Figure). PDD and moderate to severe depression symptoms were defined using *International Classification of Diseases, Ninth Revision* and *International Statistical Classification of Diseases and Related Health Problems, Tenth Revision* codes and a Patient Health Questionnaire–9 (PHQ-9) score of 10 or higher,^4^ respectively, during pregnancy. Undiagnosed depression was identified as a PHQ-9 score of 10 or higher without evidence of depression diagnosis.

**Figure.**
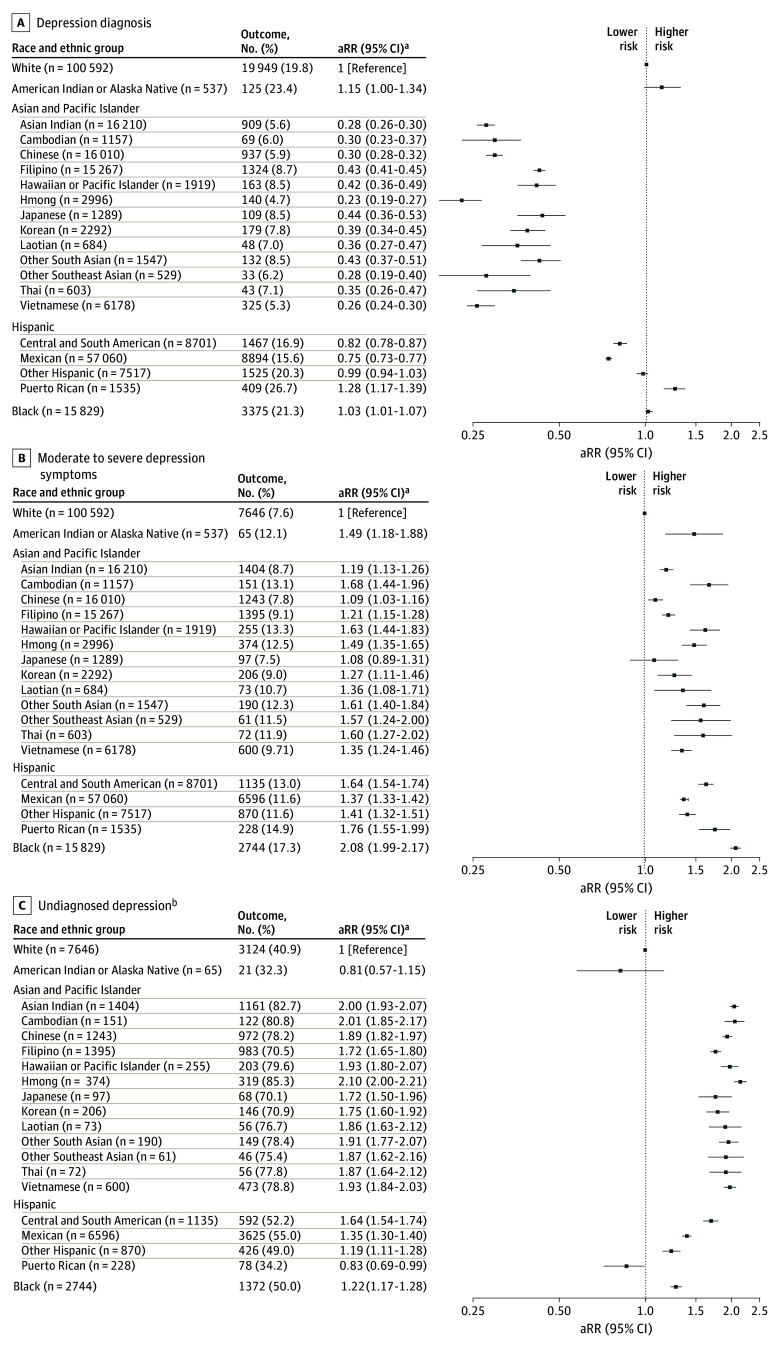
Risk of Prenatal Depression Diagnosis, Moderate to Severe Depression Symptoms, and Undiagnosed Depression Error bars represent 95% CIs. aRR indicates adjusted relative risk. ^a^Adjusted for maternal age, parity, and delivery year. ^b^Among sample with moderate to severe depression.

Modified Poisson regression models with robust SEs were used to estimate relative risk (RR) of each study outcome associated with racial and ethnic subgroups, adjusted for a priori–specified confounders (maternal age, parity, delivery year). Analyses were conducted from December 2023 to August 2024, using SAS 9.4 (SAS Institute).

## Results

Among 258 452 participants (maternal mean [SD] age, 30.7 [5.3] years), PDD prevalence was 15.5% and prevalence of moderate to severe depressive symptoms was 10.9% (Table). Within subgroups, prevalence ranged from 4.7% (Hmong) to 26.7% (Puerto Rican) for PDD and from 7.5% (Japanese) to 17.3% (Black) for symptoms (Figure). Compared with White individuals, Puerto Rican (adjusted RR [aRR], 1.28; 95% CI, 1.17-1.39) and Black (aRR, 1.03; 95% CI, 1.01-1.07) individuals had significantly higher risk of PDD. Significantly lower PDD risk was observed among Asian and Pacific Islander (Vietnamese: aRR, 0.25; 95% CI, 0.24-0.30), Mexican (aRR, 0.75; 95% CI, 0.73-0.77), and Central and South American (aRR, 0.82; 95% CI, 0.78-0.87) individuals compared with White individuals (Figure).

**Table.** Characteristics of Pregnant Individuals in Kaiser Permanente Northern California between January 1, 2013, and December 31, 2019

Characteristic	Participants, No. (%) (N = 258 452)
Race and ethnicity	
American Indian or Alaska Native	537 (0.2)
Asian and Pacific Islander	
Asian Indian	16 210 (6.3)
Cambodian	1157 (0.5)
Chinese	16 010 (6.2)
Filipino	15 267 (5.9)
Hawaiian or Pacific Islander	1919 (0.7)
Hmong	2996 (1.2)
Japanese	1289 (0.5)
Korean	2292 (0.9)
Laotian	684 (0.3)
Other South Asian^a^	1547 (0.6)
Other Southeast Asian^a^	529 (0.2)
Thai	603 (0.2)
Vietnamese	6178 (2.4)
Hispanic	
Central and South American	8701 (3.4)
Mexican	57 060 (22.1)
Other Hispanic^a^	7517 (2.9)
Puerto Rican	1535 (0.6)
Black	15 829 (6.1)
White	100 592 (39.0)
Prenatal depression diagnosis^b^	
Yes	40 155 (15.5)
No	218 297 (84.5)
Depression severity^c^	
None to mild	207 384 (80.2)
Moderate to severe	25 405 (10.9)
Missing data	25 663 (9.9)
Maternal age, y	
15-24	38 702 (15.0)
25-29	70 894 (27.4)
30-34	93 056 (36.0)
35-45	55 800 (21.6)
Educational level	
<High school diploma	7501 (2.9)
High school diploma or GED	39 845 (15.4)
Some college	72 145 (27.9)
Bachelor’s degree	68 762 (26.6)
Postgraduate	43 894 (17.0)
Missing data	26 305 (10.2)
Parity	
0	113 684 (44.0)
1	89 411 (34.6)
≥2	53 705 (20.8)
Missing data	1652 (0.6)
Medicaid	
No	233 336 (90.3)
Yes	25 116 (9.7)
NDI quartile	
1 (least deprived)	64 793 (25.1)
2	64 578 (25.0)
3	64 326 (24.9)
4 (most deprived)	63 882 (24.7)
Missing data	873 (0.3)
Delivery year	
2013	32 804 (12.7)
2014	34 665 (13.4)
2015	36 484 (14.1)
2016	37 855 (14.7)
2017	38 083 (14.7)
2018	38 349 (14.8)
2019	40 212 (15.6)

Abbreviations: GED, General Educational Development; NDI, Neighborhood Deprivation Index.

^a^ Other South Asian ethnicities included Pakistani, Nepalese, Sri Lankan, and Bangladeshi or Bengali. Other Southeast Asian ethnicities included Malaysian, Indonesian, Singaporean, and Burmese. Other Hispanic included ethnic groups in countries where the primary spoken language is Spanish.

^b^ Prenatal depression diagnosis was documented between the first day of the last menstrual period through the day before a live birth.

^c^ Depression severity was measured by Patient Health Questionnaire–9 (PHQ-9) and documented between the first day of the last menstrual period through the day before a live birth. PHQ-9 was administered to pregnant individuals as part of KPNC’s standard prenatal care at the first prenatal visit and at 26 to 28 weeks.^3^ None to mild depression symptoms were defined as PHQ-9 score of 0 to 9, and moderate to severe depression symptoms were defined as PHQ-9 score of 10 or higher; higher scores indicated more symptoms. A PHQ-9 score of 10 or higher is consistent with the clinical threshold for a depression diagnosis.^4^

Risk of moderate to severe depressive symptoms was significantly greater among all groups compared with White individuals, with increased risk ranging from 9% (Chinese: aRR, 1.09; 95% CI, 1.03-1.16) to 108% (Black: aRR, 2.08; 95% CI, 1.99-2.17) (Figure). Risk of undiagnosed depression was significantly higher across all groups with moderate to severe symptoms compared with White individuals, except American Indian or Alaska Native (aRR, 0.81; 95% CI, 0.57-1.15) and Puerto Rican (aRR, 0.83; 95% CI, 0.69-0.99) individuals. For Asian subgroups, aRR was generally higher than for Black or Hispanic individuals, with highest risk among Hmong individuals (aRR, 2.10; 95% CI, 2.00-2.21) (Figure).

## Discussion

The findings underscore the importance of disaggregating race and ethnicity data, especially among Asian and Hispanic populations, to better understand PDD burden and symptom severity. Heterogeneity among Hispanic and Asian groups attributed to factors such as nativity (US-born vs non-US-born) and acculturation may contribute to intragroup disparities.^5^ The results also highlight higher risk of depression underdiagnosis among racial and ethnic groups, which may precede treatment-initiation disparities. To increase engagement, future research should explore how factors such as attitudes about mental illness, lack of cultural competence in health care settings, and residential segregation contribute to disparities.

KPNC’s diverse membership and PDD screening program capturing self-reported symptom severity strengthen this study. A study limitation is that findings may not be generalizable to uninsured pregnant individuals.

## Notes

Supplement.
Data Sharing Statement
