# Supplementary material for: Prenatal Depression and Symptom Severity by Maternal Race and Ethnicity
Source: JAMA Netw Open. 2025 Mar 13;8(3):e250743. doi: 10.1001/jamanetworkopen.2025.0743 (PMC11907315; doi:10.1001/jamanetworkopen.2025.0743)
Supplement: Supplement. — Data Sharing Statement [file jamanetwopen-e250743-s001.pdf]

Data Sharing Statement

Kelly-Taylor. Prenatal Depression and Symptom Severity by Maternal Race and Ethnicity. *JAMA Netw Open*. Published March 13, 2025. doi:10.1001/jamanetworkopen.2025.0743

Data
Data available: No
